# Local treatment for metastatic and primary sites in metastatic renal cell carcinoma in the combination immunotherapy era: a narrative review

**DOI:** 10.1007/s10147-026-02982-8

**Published:** 2026-02-13

**Authors:** Junya Abe, Takaya Murashima, Shimpei Kojima, Takashi Ueno, Akinori Takei, Naoko Nakai, Takahiro Akioka, Toshiyuki Kamoto, Atsuro Sawada

**Affiliations:** Department of Urology, Faculty of Medicine, Miyazaki University Hospital, 5200 Kihara, Kiyotake, Miyazaki, 889-1692 Japan

**Keywords:** Renal cell carcinoma, Cytoreductive nephrectomy, Metastasectomy

## Abstract

Advances in immuno-oncology (IO)-based systemic therapies have improved treatment outcomes for patients with metastatic renal cell carcinoma (mRCC). However, long-term survival of these patients remains challenging, highlighting the need to reassess the role of local and metastasis-directed treatments. Cytoreductive nephrectomy (CN) has traditionally been a part of the therapeutic armamentarium for mRCC, and evidence from the targeted therapy era—most notably the CARMENA and SURTIME trials—indicates that deferred CN after initial systemic therapy may benefit carefully selected patients. In the IO era, prospective evidence regarding CN is lacking, although ongoing trials, such as NORDIC-SUN and PROBE, are expected to refine patient selection and optimal timing. Real-world analysis reveals a significant decline in conducting CN since 2018. However, CN remains associated with improved overall survival of patients who received several IO-based first-line regimens after adjustment for baseline characteristics. Metastasis-directed treatments, including metastasectomy and local interventions for bone metastases, continue to exhibit potential survival benefits and may maintain functional status when complete resection of lesions is achievable. Considering the absence of definitive prospective data applicable to routine clinical practice, individualized treatment strategies should consider CN and local therapies alongside systemic treatment response, tumor biology, and patient-specific prognostic factors.

Globally, renal cell carcinoma (RCC) affects more than 400,000 individuals annually and leads to over 150,000 deaths annually. These numbers are likely to continue increasing toward 2050, and the number of deaths are likely to double [1]. With the widespread implementation of screening programs and advances in imaging modalities, opportunities for detecting RCC have increased. Most cases are identified as small renal masses, with approximately 10%–15% being diagnosed as metastatic RCC (mRCC) at presentation [2]. Even in stage I RCC, approximately 20% of patients develop metastatic recurrence during a 20-year postoperative follow-up. Among these are cases of late recurrence occurring after 10 or more years of surgery [3].

Surgical intervention, including partial nephrectomy or radical nephrectomy, remains the primary treatment option for localized RCC without metastasis. Conversely, surgical resection of the primary tumor before systemic therapy, such as cytoreductive nephrectomy (CN), as well as metastasectomy, has historically been considered an important therapeutic strategy in RCC with metastasis. A meta-analysis of two randomized controlled trials, SWOG-8949 and EORTC-30947, showed the clinical benefit of CN in the cytokine era [4, 5]. Moreover, spontaneous regression of metastatic lesions after CN has been observed in some cases, and its effectiveness has been reported [6].

Various molecular targeted therapies, including tyrosine kinase inhibitors (TKI), have been successively introduced since the superior efficacy of sorafenib over interferon was demonstrated in 2007 [7]. Furthermore, immune checkpoint inhibitors (immuno-oncology [IO]) were introduced in 2016, leading to a dramatic evolution in the treatment landscape of mRCC. In particular, after the advent of combination immunotherapy that involves IO + IO or IO + TKI, the prognosis of patients with mRCC has markedly improved due to high response rates and substantial tumor reduction. Consequently, the previously demonstrated clinical benefit of CN performed before systemic therapy (upfront CN) warrants re-evaluation. Furthermore, based on international clinical trials and high-quality clinical studies, the concept of deferred CN—where CN is performed after initial systemic therapy—has become established [8, 9]. In this review, we revisit the existing evidence on CN in the post-TKI era and discuss the contemporary role and timing of CN, including deferred CN.

## Cytoreductive nephrectomy in the molecular targeted therapy era

In the CARMENA trial, patients with mRCC were randomized to receive either sunitinib monotherapy or sunitinib after upfront CN and revealed that sunitinib monotherapy was non-inferior to the combination of CN followed by sunitinib. The median overall survival (OS) was 28.4 and 13.9 months in the sunitinib-alone and upfront CN + sunitinib groups, respectively (hazard ratio [HR]: 0.89, 95% confidence interval [CI]: 0.71–1.10) [10]. Based on these findings, a reduction in the number of CN procedures has been reported in the United States [11]. However, the interpretation of the CARMENA trial results has been the subject of considerable debate. One major concern is the inclusion of a non-negligible proportion of patients (17%) in the sunitinib monotherapy group who subsequently underwent nephrectomy, thereby raising the issue of treatment crossover and potentially confounding the true impact of CN. Further, the fact that more than 40% of the enrolled patients were classified as Memorial Sloan–Kettering Cancer Center (MSKCC) poor risk has been criticized as this indicates that the study population may have included a substantial number of patients with poor prognosis and high metastatic burden, in whom the benefit of CN would not be expected. Importantly, the key message of this trial should not be interpreted as a simple recommendation to omit CN; rather, the authors themselves emphasized that the optimal patient selection for CN remains unclear. A post hoc subgroup analysis of the CARMENA trial based on International Metastatic Renal Cell Carcinoma Database Consortium (IMDC) risk classification has been reported [12]. Among patients with two or more IMDC risk factors, OS was significantly longer in those treated with sunitinib alone (31.2 months vs. 17.6 months; *p* = 0.03). Conversely, OS was numerically superior in the upfront CN group compared with the sunitinib-alone group in patients with a single IMDC risk factor, although not statistically significant (31.4 months vs. 25.2 months; *p* = 0.2). These findings are derived from post hoc analysis, but they may help clarify this unresolved clinical question discussed above (Table 1).

The SURTIME randomized clinical trial, conducted contemporaneously with the CARMENA trial, compared survival outcomes between patients with mRCC who received sunitinib after upfront CN and those who underwent deferred CN after initial treatment with sunitinib [13]. The study was underpowered due to limited patient accrual; however, a significant OS benefit was demonstrated in the deferred CN group compared with the upfront CN group (median OS: 32.4 months vs. 15.0 months; HR: 0.57, 95% CI: 0.34–0.95; *p* = 0.032). These findings indicate a potential benefit of deferred CN, the role of which remained unclear in the CARMENA trial due to its inclusion within the sunitinib monotherapy arm. Furthermore, the high proportion of patients with MSKCC intermediate-risk (88%) and the relatively small number of poor-risk patients in the SURTIME trial may serve to complement the CARMENA data. Notably, approximately 20% of patients were unable to receive systemic therapy and experienced a fatal clinical course in the upfront CN group, whereas nearly all patients in the deferred CN group underwent systemic treatment. Further, deferred CN provides the advantage of identifying patients with inherent resistance to molecular targeted therapy. These patients are estimated to account for approximately 25% of cases. These two prospective trials do not fully represent all patients with mRCC; whereas, deferred CN in the TKI era may be considered a potentially effective treatment strategy when performed with appropriate and careful patient selection.

## Cytoreductive nephrectomy in the immuno-oncology era

At present, IO-based regimens are recommended as first-line systemic therapy for patients with mRCC. To date, no prospective trials have investigated the clinical utility of CN in the IO era. Several ongoing clinical studies, including NORDIC-SUN and PROBE, are currently underway, and their results are awaited [14, 15]. Considering the accumulated evidence from the TKI era, consensus is growing that systemic therapy should be prioritized in mRCC management. Consequently, the evidence regarding CN in the IO era is presently limited to retrospective studies and large database analyses. It should be noted that these studies are methodologically limited to patients who remain fit for surgery after systemic therapy, and therefore may predominantly reflect outcomes in a biologically favorable subset rather than the true effect of surgery.

Singla et al. retrospectively analyzed data from 391 patients with mRCC who received IO therapy (ipilimumab + nivolumab) using the National Cancer Database. Among these patients, 56.5% (n = 221) received IO combined with CN, whereas 43.5% (n = 170) received IO alone. During a median follow-up of 14.7 months, patients who underwent CN demonstrated superior OS and a lower risk of mortality compared with those treated with IO alone (IO + CN: not reached vs. IO alone: 11.6 months; HR: 0.23, p < 0.001). The CN group was further categorized into an IO + deferred CN group (n = 24) and an upfront CN + IO group (n = 197). Patients in the IO + deferred CN group demonstrated lower pT stage, nuclear grade, tumor size, and rate of lymphovascular invasion. Further, the primary tumor demonstrated a pT0 status in two patients (10%) within this group. Moreover, Shirotake et al. evaluated 10 patients who underwent IO + deferred CN and revealed that no viable tumor cells were identified in the surgical specimens in three cases [16, 17].

### Optimal patient selection

Similar to the TKI era, no high-level evidence currently exists in the IO era to clearly define the optimal candidates for CN. Current guidelines do not recommend upfront CN and instead limit their recommendations to considering deferred CN in patients who showed a therapeutic response to systemic therapy [18]. An exception is made for cases in which CN is performed as a palliative intervention for symptoms originating from the primary renal lesion. The National Comprehensive Cancer Network, American Urological Association, and American Society of Clinical Oncology guidelines recommend that upfront CN should be considered only in carefully selected patients with a single IMDC risk factor in whom the majority of the tumor burden can be surgically resected [19].

Moreover, the SCREEN score has been proposed as a tool to identify patients for whom upfront CN should be avoided. Specifically, upfront CN is discouraged in patients who meet four or more of the following seven criteria: metastases involving three or more organs, maximum metastatic diameter of ≥ 5 cm, presence of bone metastasis, systemic symptoms (e.g., fever, weight loss, etc.), anemia, hypoalbuminemia, and neutrophil-to-lymphocyte ratio of > 4 [20]. However, these criteria are hypothesis-generating and derived from retrospective studies, and therefore should not be considered definitive.

Conversely, the role of CN in mRCC accompanied by tumor thrombus extension into the inferior vena cava remains controversial. In venous tumor thrombus cases, treatment decisions must consider the risk of sudden death due to pulmonary embolism caused by tumor fragments or thrombi. Even in such patients, initiating systemic therapy first may enable tumor shrinkage, thereby potentially enabling the performance of deferred CN as a less invasive, downsized surgical procedure. Conversely, tumor regression may increase the risk of embolization due to tumor thrombus detachment, requiring careful consideration. Current guidelines assign a low level of recommendation for upfront CN in patients with mRCC and venous tumor thrombus [21, 22]. At present, surgical removal of the venous tumor thrombus should remain a therapeutic option under consideration in the context of achieving long-term survival for such cases, while prioritizing systemic therapy.

### Optimal systemic therapy prior to CN

Subgroup analysis that evaluates the impact of CN have been conducted across various first-line IO regimens [23–27]. The presence of selection bias is widely recognized; however, the reported HRs for OS, ranging from 0.52 to 0.79 in favor of the CN groups, indicate a potential benefit of CN even in the IO era. In real-world clinical practice, analyses of large-scale datasets have revealed a notable decline in the use of CN from 2005 to 2024, particularly after the publication of the CARMENA trial results in 2018 [28]. This downward trend has continued even after widely adopting IO–IO and IO–TKI regimens, with only approximately 5% of patients with mRCC undergoing CN in recent years. In the same study, propensity score-matched analysis using a large national database was conducted to evaluate 5-year survival outcomes according to the performance of CN in the IO era. The CN groups demonstrated significantly longer OS compared with the non-CN groups across all four first-line IO-based regimens evaluated (pembrolizumab + axitinib, nivolumab + cabozantinib, pembrolizumab + lenvatinib, and nivolumab + cabozantinib). These results indicate that even in the IO era, selected patients who are appropriate candidates for CN may still derive a survival benefit from undergoing CN. However, because this retrospective study included only a highly selected group of patients undergoing CN, confounding remains significant, and the survival benefit associated with CN cannot be considered definitive. Direct comparisons of treatment efficacy across individual regimens could not be performed within the above-mentioned database. However, approximately half of the patients received nivolumab + ipilimumab—which typically produces relatively early therapeutic responses—followed by nivolumab + cabozantinib, whereas pembrolizumab + lenvatinib accounted for a smaller proportion of cases. A simple cross-trial comparison is not feasible, considering the results of the first-line clinical trials described earlier. However, the HR for OS was numerically most favorable in the pembrolizumab + lenvatinib regimen [27].

### Optimal timing of CN

No clearly defined criteria regarding the optimal timing of CN are available. However, determining when to perform CN is a critical challenge, comparable in importance to patient selection. The timing of CN requires careful balancing of potential risks and benefits. Early CN after a short course of systemic therapy may reduce cumulative treatment-related toxicity. However, it also carries a risk of overtreatment, particularly in patients who may achieve complete or durable responses with systemic therapy alone, a concern that is especially relevant in the IO era. In contrast, prolonged systemic therapy may result in tumor shrinkage before surgery, potentially reducing surgical complexity and invasiveness, especially in cases with IVC thrombus. However, extended systemic treatment may also lead to resistance to first-line therapy, potentially resulting in missed opportunities for surgical intervention. Importantly, disease progression during systemic therapy may reflect intrinsic tumor biology rather than the timing of surgery itself. Therefore, prioritizing CN solely to avoid disease progression may also represent overtreatment. Overall, these considerations underscore that the optimal timing of CN involves a trade-off among potential benefit, overtreatment, and residual risk, and should be reassessed throughout the course of systemic therapy in metastatic renal cell carcinoma.

Patients in both the NORDIC-SUN and PROBE trials are randomized to undergo CN or continue systemic therapy after approximately 3 months of initial treatment. Notably, in the PROBE trial, patients whose lesions are deemed unresectable at the 3-month assessment are excluded from randomization, whereas the NORDIC-SUN trial includes an additional re-evaluation of lesion resectability after a further 12 weeks before randomization, which is a distinguishing feature of its study design.

Considering that first-line IO regimens have reported progression-free survival durations of 11.6–24 months, treatment response could be observed for a longer period before proceeding with CN. Real-world data have revealed that the median time to deferred CN from systemic therapy initiation is approximately 6 months [29]. Based on the results of ongoing phase III trials, clinicians may increasingly need to consider CN as a therapeutic option during the course of systemic therapy; however, future studies are needed to define the optimal timing of CN by incorporating the balance between potential risks and benefits.

### Local treatment of metastatic lesions

Surgical treatment for metastatic lesions in mRCC has long been performed, and long-term survivors after metastasectomy have been reported [30]. According to the 2026 European Association of Urology guidelines, surgical resection of mRCC lesions should be considered in patients who meet favorable prognostic criteria and in whom complete resection of lesions is achievable, particularly for controlling local symptoms.

No prospective trials have evaluated local treatment for metastatic lesions; however, numerous retrospective studies have been published. A systematic review that summarizes retrospective studies from the cytokine to the targeted therapy era reported significant improvements in OS or cancer-specific survival (CSS) in 6 of 8 studies, favoring metastasectomy (40.8 months vs. 14.8 months). Furthermore, the remaining two studies demonstrated a numerical improvement in survival, although the difference did not reach statistical significance [31]. This review revealed that complete metastasectomy was associated with superior OS compared with incomplete metastasectomy.

No studies have exclusively evaluated patients treated with targeted therapy alone. However, a retrospective study published in 2022 investigated patients who received either targeted therapy or IO. Complete resection was associated with a remarkably prolonged CSS of 6.1 years, significantly longer than that observed in patients treated with targeted therapy alone (2.6 years) or IO therapy (3.5 years) [32]. Further, this study reported that patients achieved a median treatment-free interval of 11.2 months before developing new metastatic lesions after complete resection.

However, all of these studies are retrospective and include a substantial proportion of patients with advanced disease who were not suitable candidates for surgery. Therefore, identifying whether the observed survival benefit is attributable to metastasectomy itself or to the underlying tumor biology that makes such surgical treatment feasible remains challenging. At minimum, when surgical cytoreduction of metastatic sites can be achieved, metastasectomy offers two potential advantages: (1) providing a treatment-free interval without systemic therapy and (2) reducing exposure to systemic treatment-related adverse events.

In the KEYNOTE-564 trial, a subgroup analysis of patients who underwent complete resection of metastatic lesions revealed a favorable HR for disease-free survival (HR: 0.29) in the adjuvant pembrolizumab group [33]. This indicates 1 year of adjuvant pembrolizumab after metastasectomy as a reasonable option. However, whether adjuvant therapy should be pursued in this setting or whether combination IO–IO or IO–TKI therapy at the time of recurrence is preferable remains unclear, as direct comparison is unavailable.

Bone is the second most common site of metastasis in RCC after the lung. Further, RCC bone metastases are highly osteolytic and carry a substantial risk of skeletal-related events (SREs), including severe pain due to pathological fractures and paraplegia caused by spinal cord compression. SREs adversely affect performance status (PS) and overall prognosis; thus, local therapies for RCC bone metastases—such as radiotherapy, palliative surgery, and administration of bone-modifying agents—are frequently selected to prevent SREs or to alleviate symptoms upon their occurrence.

Fuchs et al. compared outcomes between patients with solitary RCC bone metastases at various anatomical sites who underwent metastasectomy and/or curettage with local stabilization versus those who received no local treatment [34]. They revealed that the intervention group demonstrated a significantly higher 5-year CSS. Multivariate analysis adjusted for prior nephrectomy status, age, and sex revealed that metastasectomy/curettage with stabilization remained a significant, favorable prognostic factor.

Complete resection of RCC bone metastases may be meaningful in selected patients even in the IO era. In particular, surgical resection may provide not only long-term maintenance of activities of daily living and quality of life but also the potential for improved survival when complete removal is achievable for bone metastases that markedly impair PS through SREs.

## Conclusion

**Fig. 1:**
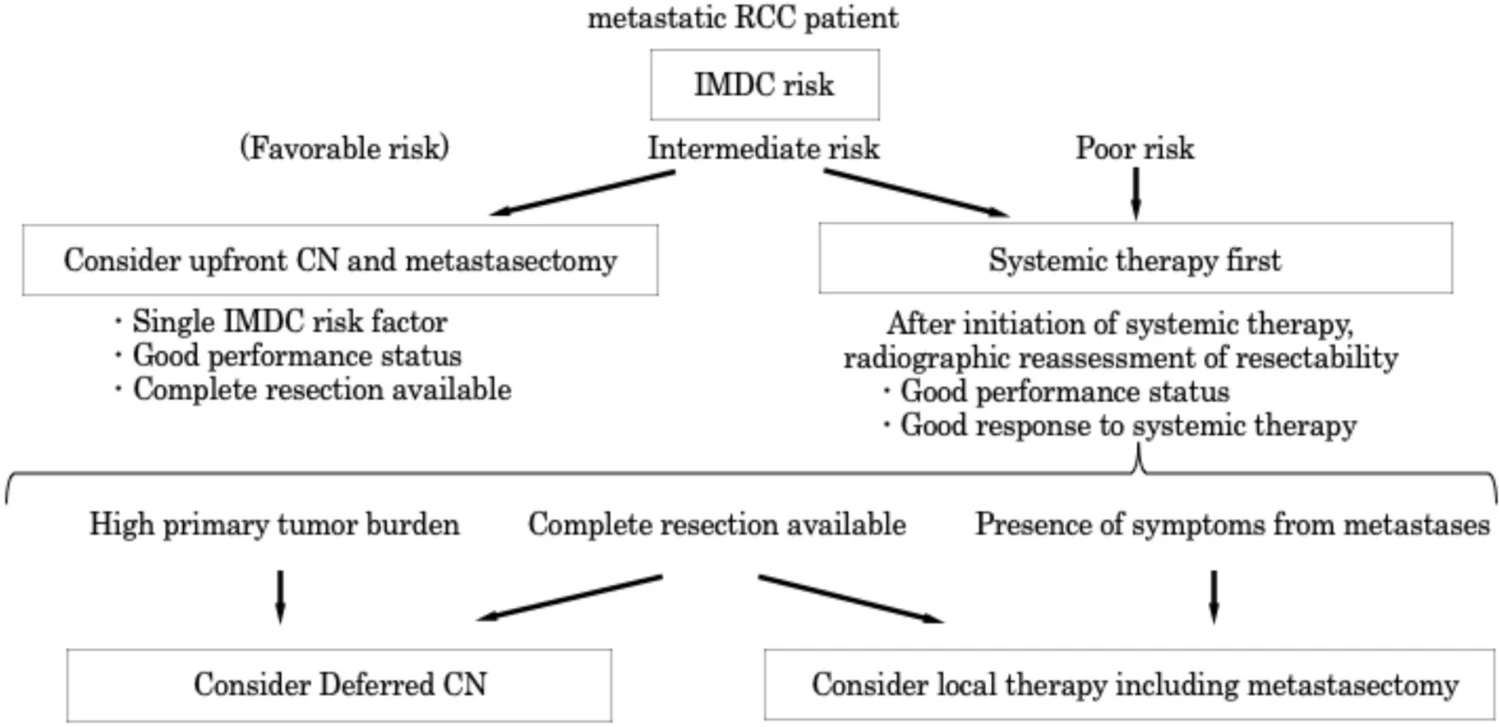
Treatment algorithm for metastatic renal cell carcinoma. This figure presents the treatment algorithm proposed by the authors. RCC, renal cell carcinoma; IMDC, International Metastatic Renal Cell Carcinoma Database Consortium; CN, cytoreductive nephrectomy

Treatment outcomes for mRCC have improved in recent years with the introduction of IO-based systemic therapies; however, long-term survival remains limited for many patients. Concurrently, selected individuals are well recognized to achieve prolonged survival through classical local or metastasis-directed interventions. We present a simplified treatment algorithm in Fig. 1 illustrating when CN and local therapy may be considered during systemic therapy. Despite several ongoing clinical trials, prospective evidence that closely reflects real-world clinical practice remains limited. At present, there is no definitive evidence to answer the fundamental clinical questions of which patients should undergo CN and when it should be performed, and establishing such evidence remains inherently challenging. The studies discussed in this manuscript are summarized in Table 1. To distinguish biological responders from those who truly benefit from surgery, appropriately designed randomized controlled trials are needed to overcome selection bias and to clarify the optimal timing of cytoreductive nephrectomy. Considering the limitations of current evidence, clinicians must continue to consider local or metastasis-directed treatment options when selecting the most appropriate, individualized therapeutic strategy for each patient.

**Table 1:** Summary of studies in metastatic renal cell carcinoma

	CARMENA [10]	SURTIME [13]	Singla et al.[16]	Nukaya el al. [29]	Manivasagam et al.[28]	PROBE [14]	NORDIC-SUN [15]
trial duration	2009–2017	2010–2016	2015–2016	2018–2023	2005–2024	2021–2033	2023–2026
published	2018	2019	2020	2025	2025	not yet	not yet (2029)
design	RCT	RCT	database	retrospective	database	RCT	RCT
treatment era	TKI	TKI	IO	IO	cytokine-IO	IO	IO
CN timing	upfront	both	both	both	not mentioned	deferred	deferred
primary endpoint	OS	PFS	OS	not mentioned	OS	OS	OS

RCT, randomized controlled trial; TKI, tyrosine kinase inhibitor; OS, overall survival; PFS, progression-free survival; IO, immuno-oncology

This table summarizes the key studies and data sources discussed in this manuscript
